# Resilience is inversely associated with self-harm behaviors among Chinese adolescents with childhood maltreatment

**DOI:** 10.7717/peerj.9800

**Published:** 2020-09-08

**Authors:** Xin Tian, Guangya Yang, Linling Jiang, Runxu Yang, Hailiang Ran, Fujia Xie, Xiufeng Xu, Jin Lu, Yuanyuan Xiao

**Affiliations:** 1 School of Public Health, Kunming Medical University, Kunming, China; 2 The First Affiliated Hospital, Kunming Medical University, Kunming, China; 3 Lincang Psychiatry Hospital, Lincang, China

**Keywords:** Self-harm, Childhood maltreatment, Resilience, Emotion regulation, Interpersonal assistance, Goal concentration

## Abstract

**Background:**

Abundant studies have identified the association between childhood maltreatment and self-harm (SH), but little has been discussed with regard to the role of resilience in SH behaviors of adolescents who had experienced childhood maltreatment. In this study, we investigated if resilience, as well as its five dimensions, could present negative associations with presence, repetition, and severity of SH among maltreated and neglected adolescents in China.

**Methods:**

A cross-sectional survey including 2,084 maltreated teenagers aged from 10 to 17 years was conducted in southwest China Yunnan province. The Childhood Trauma Questionnaire (CTQ), The Resilience Scale for Chinese Adolescents (RSCA), and the Modified version of Adolescents Self-Harm Scale (MASHS) were adopted to measure childhood maltreatment experiences, psychological resilience, and SH behaviors of the respondents, respectively. Binary univariate and multivariate logistic regression models were employed to discuss the associations between resilience and occurrence, repetition, severity of SH.

**Results:**

Among the participants who met the criteria of CTQ, the prevalence rates of SH were 63.83%, 73.94%, 71.50%, 55.53%, and 58.21% for physical abuse (PA), emotional abuse (EA), sexual abuse (SA), physical neglect (PN), and emotional neglect (EN). Final regression model demonstrated that resilience was in general inversely associated with SH, repeated SH, and severe SH for all types of childhood maltreatment, with adjusted odds ratios (aORs) ranging from 0.29 (95% CI: 0.19-0.44) to 0.46 (95% CI: 0.26-0.81). Of the five dimensions of resilience, emotion regulation served as the strongest associated factor of SH among abused youths, regardless of maltreatment types. Besides, compared with those who had lower level of goal concentration and interpersonal assistance, subjects with higher resilience level reported significantly decreased risks of SH occurrence, SH repetition, and more severe SH, in adolescents who had experienced EA and PN.

**Conclusions:**

Resilience showed inverse association with childhood maltreatment related SH in Chinese adolescents. These findings preliminarily indicated that interventions targeting on building up resilience, especially enhancing emotion regulation ability, improving goal concentration, and consolidating interpersonal assistance, could be effective in reducing SH risk, repetition, and severity in maltreated Chinese teenagers.

## Introduction

Self-harm (SH) is most frequently reported in adolescents, with a prevalence three times higher than which in adults (Ogle & Clements, 2008). In China, a multi-center study estimated that juveniles who had ever engaged in SH were nearly one-third of all enrolled study subjects (Xin et al., 2017). It has been consistently verified that SH is one of the strongest predictors of future suicide (Chan et al., 2016). In fact, SH predisposes teenagers a seven-fold risk of suicide attempts, even after controlling for other established risk factors like suicide attempt history and baseline depression (Cox et al., 2012). Published studies revealed that, among those who relinquished their lives, up to 50% had been involved in SH behaviors in the past, and SH repetition appears to be more common in the following months of the index episode (Foster et al., 1999; Owens, Horrocks & House, 2002). Given its omnipresence in adolescents, as well as the intimate association with suicidal behaviors, SH should be effectively intervened in youngsters.

Childhood maltreatment refers to any abuse or neglect behaviors with detrimental effects on children’s health, survival, esteem, or development (World Health Organization, 2016). It can be classified into five types: physical abuse (PA), emotional abuse (EA), sexual abuse (SA), physical neglect (PN), and emotional neglect (EN) (Gilbert et al., 2009). Child abuse has long been regarded as a prime health problem due to its high occurrence. In China, the reported prevalence rates for PA, EA, SA, and neglect were 26.6%, 19.6%, 8.7%, and 26.0%, respectively, based on a meta-analysis (Fang et al., 2015). Childhood abuse is recognized as a salient risk factor for SH, and evidence from both population and clinical studies have revealed a close relationship between SA, EA, neglect and SH in late adolescence (Lang & Sharma-Patel, 2011; Hawton, Saunders & O’Connor, 2012). Another large sample study conducted in mainland China middle schools showed that, those who were found at a higher risk of SH tended to be victims of childhood maltreatment, irrespective of the specific type of events (Wan et al., 2015). Thus, investigations on what may serve as protective factors of SH among this at-risk young group are of important significance for targeted interventions.

More general, resilience is interpreted as desirable adaptation competence of an individual when suffering from adverse experiences (Luthar, 2003). Research on the association between SH and resilience has revealed that, except for buffering against deterioration from self-injury to suicidal behaviors, resilience could also protect against SH (Nagra, Lin & Upthegrove, 2016). In a youth-centric survey, people living with SH and violence presented significantly lower resilience than their counterparts (Huang & Mossige, 2015). Moreover, the favorable role of resilience in SH is strengthened by our preceding study in Chinese left-behind children (Tian et al., 2019). Despite seen as high-risk group, a large proportion of maltreated adolescents will not develop SH (Holmes et al., 2015). In this regard, it is possible that mistreated juveniles who engaged in SH might be less resilient. Consequently, resilience may be protective for abused adolescents in antagonizing SH.

Although some published studies concluded that emotion dysregulation, an important dimension of resilience, was associated with SH behaviors in mistreated juveniles, their limited sample size and hospital-based survey design hampered the validity of study results (Chaplo et al., 2015; Peh et al., 2017; Titelius et al., 2018). Moreover, studies which addressed the association between other major dimensions of resilience (goal concentration, interpersonal assistance, positive perception, family support) and SH among maltreated adolescents are still scarce. Aside from SH incidence, the possible protective role of resilience in SH severity and repetition for abused teenagers invites investigation but has seldomly been discussed.

With regard to the shortcomings stated above, in the present study, by using data collected from a population-based cross-sectional design, we intended to examine the hypothesized positive role of resilience, as well as its five dimensions, in SH of adolescents who had exposed to different types of childhood maltreatment. We put forward the assumption that resilience is negatively associated with SH occurrence, severity, and repetition among victims of childhood abuse.

## Methods

### Design and procedure

Following the approval from the Ethics Committee of The Third People’s Hospital of Lincang (Lincang Psychiatry Hospital), a cross-sectional study was conducted from December 1 to 13, 2019, in Lincang municipality, Yunnan Province, China. The major reason for choosing Lincang as our study site is that, this city has been participating in a national program which aiming at improving mental health of the general public, therefore, it has solid basis for population-based psychological survey and the possible subsequent intervention study. Lincang bordering Myanmar on its southwest, belongs to subtropical region, with an east longitude of 98°40′–100°32′ and a north latitude of 23°05′–25°03′. By the end of 2016, Lincang had a population of 2.52 million.

Participants were selected using a three-stage simple random clustering sampling design: (1) In the first stage, Linxiang district was randomly chosen among all 8 districts and counties within Lincang’s jurisdiction; (2) Fourteen schools (5 primary schools, 5 junior high schools, 4 senior high schools) were randomly chosen in the second stage; (2) In the third stage, within each chosen school, based on the required sample size, 3 to 4 classes were randomly selected, and all eligible students within the chosen class were potential study participants. Adolescents were further included in this analysis if they: (1) Reported any type of childhood maltreatment experience; (2) Provided complete information on critical variables (childhood maltreatment, SH behaviors, resilience, and school bullying). Besides, because in this study we simultaneously measured suicidal ideation and behaviors of the participants, and it has been suggested that only children above 10 can totally understand the definition and consequence of suicide (Mishara, 1999), therefore, we only included potential study participants whose age was above 10 and below 18. Exclusion criteria below were employed to further screen subjects: (1) Illiterate; (2) Incapable of expression; (3) Hearing dysfunction; (4) Severely ill or inconvenient to participate; (5) Refuse to participate.

## Measures

After written consents were obtained simultaneously from the legal guardian and the participant, a self-administered questionnaire survey was conducted. To guarantee the completeness of the information, upon completion, every questionnaire was reviewed carefully by pre-trained quality control personnel deployed at the survey sites. The questionnaire was comprehensive, mainly contains the following information: general characteristics, childhood maltreatment, SH behaviors, resilience, school bullying, suicidal ideation and attempts, etc. The analyses of the current study were based on the former four parts.

### Childhood maltreatment

The 28-item Childhood Trauma Questionnaire (CTQ) is a well-validated instrument which retrospectively assesses five major types of childhood maltreatment (PA, EA, SA, PN, EN) (Bernstein et al., 2003). Respondents are required to choose answers from a five-point Likert scale ranging from “never true” (1 point) to “very often true” (5 points), with a higher score represents a more serious child abuse exposure. In CTQ, each type of abuse was assessed by 5 items, therefore, the combined scores for 5 subscales of the CTQ range from 5 to 25. The recommended thresholds for PA, EA, SA, PN, and EN were: 8, 9, 6, 8, and 10, respectively (Bernstein & Fink, 1998). Participants who met the cut-off value of any type of abuse were defined as had been maltreated. In further analysis, we used the medians of subscales (9, 10.5, 7, 9, 13 for PA, EA, SA, PN, EN) to dichotomize study participants into “severe child abuse exposure” (defined as the combined score >P_50_) and “not severe child abuse exposure” (defined as the combined score ≤ P_50_). The Chinese version of the CTQ yields satisfactory internal consistency (Cronbach’s *α*: 0.78–0.90) and test-retest reliability (Kappa: 0.79–0.88) (Han et al., 2018). The Cronbach’s *α* for the CTQ in the present study was 0.81 (Bootstrap 95% CI [0.80–0.83]).

### Self-harm

We used the Modified version of Adolescents Self-Harm Scale (MASHS), a self-report instrument on the frequency and severity of the 18 most common SH behaviors in Chinese adolescents (Feng, 2008), to measure SH of the participants. A four-point Likert-type response was employed to assess the frequency of lifetime SH (0 = never; 1 = once; 2 = two to four times; 3=five times and above), and the severity of SH is measured from “non-observable injury” to “critical injury”.

### Resilience

Resilience of the participants was gauged by the Resilience Scale for Chinese Adolescents (RSCA) (Hu & Gan, 2008), a self-rated questionnaire contains 28 items with responses from “totally disagree” (1 point) to “totally agree” (5 points). RSCA can be further divided into 5 dimensions, measuring goal concentration, interpersonal assistance, emotion regulation, positive perception, and family support, respectively. The level of resilience is evaluated by the sum score of RSCA, which ranging from 28 to 140, with a higher score indicates a better resilience. The Cronbach’s *α* for RSCA in our sample was 0.86 (Bootstrap 95% CI [0.85–0.87]).

### Bullying victimization

Bullying victimization was measured by the Chinese version of Olweus Bully/Victim Questionnaire (OBVQ) (Olweus, 1996), in which the 7-item “being bullied” part was used to screen bullying victims, with two items assessing physical bullying, two items measuring verbal bullying, two items measuring relational bullying, and a single item measuring other forms of bullying. All subjects were asked to choose the frequency of events described in the past 12 months from the following responses: never (1 point), once or twice in total (2 point), once or twice in a month (3 point), once in a week (4 point), more than once in a week (5 point). Bullying victimization was defined as the score to any item is no less than 3.

### Data analysis

All data were analyzed by using R software (Version 3.6.2, The R Foundation for Statistical Computing, Vienna, Austria), the “survey” package was used in order to adjust for unequal sampling probability caused by clustering sampling design. Descriptive statistics were calculated to describe and compare general features of the study subjects. Univariate and multivariate binary Logistic regression models which taking the presence of SH as the dependent variable were then performed sequentially for PA, EA, SA, PN, and EN exposed subjects. Then, binary Logistic regression analyses exclusively for the subgroup of self-harmed participants were conducted to identify associated factors of SH repetition and severity among mistreated adolescents. Multivariate models which incorporated the five dimensions of resilience were fitted subsequently to discuss their associations with SH in maltreated youths. Statistical significance was set as a two-tailed probability less than 0.05, however, for univariate analysis, a less strict criterion of *p* < 0.1 was chosen for selection of possible covariates.

## Results

### Descriptive statistics

A total of 3,241 questionnaires were collected, among them, 7 failed quality check, 88 were excluded because of ineligible age (below 10 or above 18 years), leaving 3146 valid for further analysis, with a response rate of 97.07%. By using CTQ, 2,084 adolescents reported had experienced at least one type of childhood maltreatment, among them, 517, 706, 407, 1,167, and 1,395 were PA, EA, SA, PN, and EN victims, respectively. The demographic and socioeconomic characteristics of study subjects were presented in Table 1.

**Table 1:** General characteristics of study subjects by different types of childhood maltreatment.

**Factors**	**Anymaltreatment (*N*=2084)**	**PA (*N*=517)**	**EA (*N*=706)**	**SA (*N*=407)**	**PN (*N*=1167)**	**EN (*N*=1395)**
Gender (*N*, %): Boys	1009 (48.42%)	312 (60.35%)	265 (37.54%)	228 (56.02%)	598 (51.24%)	664 (47.60%)
Age (Mean ± SD)	13.28 ± 2.20	12.80 ± 2.12	13.64 ± 2.14	13.45 ± 2.22	13.19 ± 2.18	13.30 ± 2.21
Ethnicity (*N*, %): Han	1439 (69.05%)	375 (72.53%)	461 (65.30%)	283 (69.53%)	820 (70.27%)	980 (70.25%)
Grade (*N*, %)						
Primary school	788 (37.81%)	229 (44.29%)	216 (30.59%)	127 (31.20%)	473 (40.53%)	525 (37.63%)
Middle school	688 (33.01%)	184 (35.59%)	232 (32.86%)	151 (37.10%)	385 (32.99%)	467 (33.48%)
High school	608 (29.17%)	104 (20.12%)	258 (36.54%)	129 (31.70%)	309 (26.48%)	403 (28.89%)
Place of residence (*N*, %)						
Urban	946 (45.26%)	265 (51.26%)	311 (44.05%)	149 (36.61%)	533 (45.67%)	610 (43.73%)
Rural	1138 (54.61%)	252 (48.74%)	395 (55.95%)	258 (63.39%)	634 (54.33%)	785 (56.27%)
Boarding student (*N*, %): Yes	879 (42.18%)	257 (49.71%)	456 (64.59%)	260 (63.88%)	648 (55.53%)	802 (57.49%)
Single child (*N*, %): Yes	557 (26.73%)	144 (27.85%)	186 (26.35%)	104 (25.55%)	311 (26.65%)	385 (27.60%)
Living situation (*N*, %)						
With both parents	1592 (76.39%)	390 (75.44%)	510 (72.24%)	316 (77.64%)	872 (74.72%)	1050 (75.27%)
With others^a^	492 (23.61%)	127 (24.56%)	196 (27.76%)	91 (22.36%)	295 (25.28%)	345 (24.73%)
Age of father (Mean ± SD)	42.23 ± 5.24	41.53 ± 5.18	42.42 ± 5.20	42.38 ± 5.65	42.18 ± 5.44	42.14 ± 5.28
Age of mother (Mean ± SD)	39.33 ± 4.91	39.83 ± 5.10	39.49 ± 4.85	39.57 ± 5.37	39.15 ± 4.54	39.22 ± 4.92
Educational level of father						
Primary school and below	632 (30.33%)	144 (27.85%)	226 (32.01%)	146 (35.87%)	349 (29.91%)	433 (31.04%)
Middle school	671 (32.20%)	181 (35.01%)	223 (31.59%)	131 (32.19%)	379 (32.48%)	467 (33.48%)
High school or equivalent and above	542 (26.01%)	137 (26.50%)	185 (26.20%)	87 (21.38%)	309 (26.48%)	338 (24.23%)
Unknown and missing	239 (11.47%)	55 (10.64%)	72 (10.20%)	43 (10.57%)	130 (11.14%)	157 (11.25%)
Educational level of mother						
Primary school and below	763 (36.61%)	176 (34.04%)	273 (38.67%)	180 (44.23%)	422 (36.16%)	528 (37.85%)
Middle school	635 (30.47%)	165 (31.91%)	202 (28.61%)	118 (28.99%)	363 (31.11%)	430 (30.82%)
High school or equivalent and above	504 (24.18%)	139 (26.89%)	180 (25.50%)	79 (19.41%)	286 (24.51%)	308 (22.08%)
Unknown and missing	182 (8.73%)	37 (7.16%)	51 (7.22%)	30 (7.37%)	96 (8.23%)	129 (9.25%)
Drinking (*N*, %): Yes						
Yes	587 (28.17%)	170 (32.88%)	288 (40.79%)	165 (40.54%)	339 (29.05%)	418 (29.96%)

**Notes.**

a Others include: single parent, grandparents, maternal relatives, paternal relatives, siblings, cousins, classmates, foster parents

**Figure 1:**
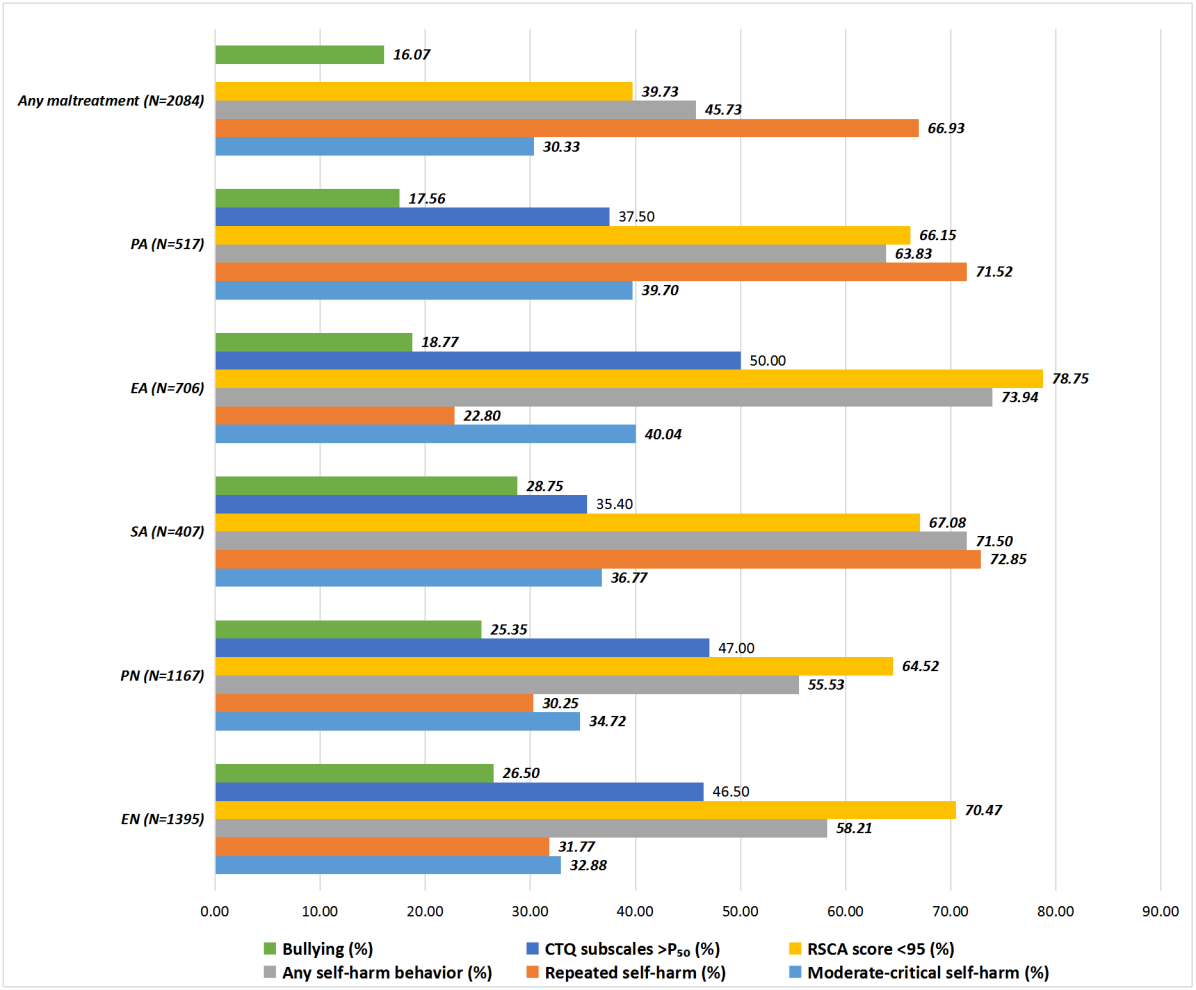
Key features of study subjects. CTQ, Childhood Trauma Questionnaire; PA, Physical abuse; EA, Emotional abuse; SA, Sexual abuse; PN, Physical neglect; EN, Emotional neglect.

Altogether 335 (16.07%) abused adolescents were classified as bullying victims and the proportion ranges from 17.56% in EN to 28.75% in SA. Subjects were further dichotomized by using the medians of the CTQ subscale scores into ≤P_50_ and >P_50_ subgroups. A total of 1131 (54.27%) mistreated adolescents reported SH behaviors, and 330 (63.83%), 522 (73.94%), 291 (71.50%), 648 (55.53%), 812 (58.21%) in PA, EA, SA, PN, and EN groups deliberately harmed themselves for at least once. Among the self-injurers, the proportions of repeated and moderate to critical SH were 66.93% and 30.33%. When classified by the median of RSCA score (95), totally 1256 (60.27%) individuals were grouped into low resilience category and the counts of less resilient youths in PA, EA, SA, PN, and EN groups were 342 (66.15%), 556 (78.75%), 273 (67.08%), 753 (64.52%), and 983 (70.47%), respectively (Fig. 1). The mean of combined RSCA score for all subjects was 90.89 (SD = 16.92), ranging from 82.79 (SD = 16.40) for EA to 88.79 (SD = 17.49) for PN.

### Association between resilience and SH in maltreated youths

Univariate binary Logistic regressions examining variables potentially influencing SH among child abuse victims were fitted (Table 2). Resilience was found to be significantly associated with SH among all abused samples in univariate analysis. After controlling for identified influencing factors, the relation between resilience and SH stayed prominent: compared to less resilient adolescents, the ORs for engaging in SH behaviors were 0.39 (95% CI [0.27–0.58]), 0.32 (95% CI [0.20–0.50]), 0.46 (95% CI [0.26–0.81]), 0.30 (95% CI [0.20–0.47]), and 0.33 (95% CI [0.20–0.55]) for adolescents of higher resilience level in PA, EA, SA, PN, and EN groups, respectively (Table 3). The ORs with their 95% CIs for influencing factors were provided in supplementary file (Table S1).

**Table 2:** Univariate Logistic regression models fitting results for SH by different types of childhood maltreatment.

Covariates	PA (*N* = 517)	EA (*N* = 706)	SA (*N* = 407)	PN (*N* = 1167)	EN (*N* = 1397)
	OR (90% CI)	OR (90% CI)	OR (90% CI)	OR (90% CI)	OR (90% CI)
Gender: Girls	2.16 (1.46–3.20)	1.54 (1.24–1.90)	1.16 (0.88–1.54)	1.78 (1.42–2.22)	1.64 (1.35-1.98)
Age: +1 year	1.40 (1.26–1.56)	1.19 (1.11–1.29)	1.36 (1.24–1.49)	1.26 (1.14–1.38)	1.28 (1.18-1.38)
Ethnicity: Han	0.97 (0.71–1.33)	1.03 (0.81–1.30)	1.38 (0.98–1.95)	1.04 (0.69–1.56)	1.18 (0.82-1.69)
Grade					
Middle school	2.21 (1.08–4.50)	1.95 (1.19–3.18)	2.60 (1.56–4.34)	2.54 (1.83–3.54)	2.44 (1.70-3.50)
High school	5.45(2.92–10.20)	2.28 (1.49–3.51)	3.75 (2.44–5.78)	3.54 (2.01–6.25)	4.00 (2.44-6.55)
Place of residence: Rural	1.92 (1.33–2.77)	1.43 (1.02–1.99)	1.40 (0.74–2.63)	1.50 (1.18–1.91)	1.68 (1.36-2.07)
Boarding student: Yes	2.52 (1.57–4.05)	1.86 (1.23–2.80)	2.51 (1.61–3.93)	2.49 (1.78–3.48)	2.69 (2.03-3.56)
Single child: No	1.00 (0.62–1.61)	1.06 (0.76–1.47)	0.96 (0.56–1.65)	1.25 (1.00–1.57)	1.19 (0.93-1.52)
Living situation: With others	1.04 (0.94–1.46)	1.60 (1.10–2.32)	0.87 (0.56–1.35)	1.46 (1.11–1.91)	1.43 (1.25-1.63)
Age of father: +1 year	1.01 (0.99–1.03)	0.99 (0.97–1.02)	1.03 (0.99–1.07)	1.03 (1.01–1.05)	1.03 (1.02-1.05)
Age of mother: +1 year	1.00 (0.97–1.02)	0.99 (0.97–1.01)	1.02 (1.00–1.05)	1.05 (1.02–1.07)	1.04 (1.02-1.07)
Educational level of father					
Middle school	1.09 (0.61–1.97)	1.08 (0.58–2.01)	1.11 (0.57–2.17)	1.13 (0.89–1.43)	1.25 (1.00-1.55)
High school or equivalent and above	0.75 (0.37–1.51)	0.67 (0.41–1.11)	0.87 (0.29–2.59)	0.81 (0.51–1.26)	0.95 (0.58-1.57)
Educational level of mother					
Middle school	0.86 (0.53–1.40)	0.99 (0.77–1.27)	0.71 (0.44–1.14)	0.75 (0.62–0.90)	0.85 (0.67-1.07)
High school or equivalent and above	0.55 (0.38–0.79)	0.54 (0.37–0.80)	0.44 (0.22–0.91)	0.65 (0.42–1.00)	0.72 (0.47-1.12)
Alcohol drinking: No	0.17 (0.10–0.31)	0.29 (0.12–0.72)	0.24 (0.12–0.50)	0.21 (0.16–0.29)	0.22 (0.14-0.34)
Bullying victimization: Yes	1.68 (1.01–2.79)	1.42 (1.00–2.03)	1.38 (0.82–2.34)	2.56 (1.67–3.91)	2.57 (1.60-4.14)
Childhood abuse severity: Moderate or above	1.81 (1.33–2.48)	2.48 (1.76–3.49)	0.81 (0.45–1.48)	1.80 (1.46–2.22)	1.44 (1.18-1.77)
Resilience: High	0.31 (0.25–0.38)	0.26 (0.17–0.41)	0.39 (0.26–0.59)	0.23 (0.17–0.31)	0.25 (0.20-0.32)

**Notes.**

CTQ Childhood Trauma Questionnaire PA Physical abuse EA Emotional abuse SA Sexual abuse PN Physical neglect EN Emotional neglect

**Table 3:** Multivariate Logistic regression models fitting results for dimensions of resilience with SH by different types of childhood maltreatment.

Types of child abuse		Resilience	Goal concentration	Interpersonal assistance	Emotion regulation	Positive perception	Family support
PA (*N* = 517)	Model 1	0.39 (0.27–0.58)	–	–	–	–	–
	Model 2	–	0.72 (0.44–1.16)	0.77 (0.51–1.18)	0.39 (0.26–0.58)	1.07 (0.62–1.84)	0.72 (0.43-1.20)
EA (*N* = 706)	Model 1	0.32 (0.20–0.50)	–	–	–	–	–
	Model 2	–	0.39 (0.29–0.53)	0.65 (0.41–1.02)	0.35 (0.20–0.60)	1.06 (0.76–1.48)	0.94 (0.63-1.40)
SA (*N* = 407)	Model 1	0.46 (0.26–0.81)	–	–	–	–	–
	Model 2	–	0.52 (0.35–0.75)	1.01 (0.53–1.90)	0.37 (0.19–0.71)	1.89 (1.05–3.41)	1.03 (0.51-2.05)
PN (*N* = 1167)	Model 1	0.30 (0.20–0.47)	–	–	–	–	–
	Model 2	–	0.67 (0.48–0.94)	0.49 (0.33–0.72)	0.42 (0.27–0.64)	1.01 (0.60–1.69)	0.90 (0.57-1.42)
EN (*N* = 1395)	Model 1	0.33 (0.20–0.55)	–	–	–	–	–
	Model 2	–	0.85 (0.60–1.22)	0.62 (0.43–0.90)	0.36 (0.26–0.51)	0.95 (0.59–1.52)	0.63 (0.49-0.82)

**Notes.**

CTQ Childhood Trauma Questionnaire PA Physical abuse EA Emotional abuse SA Sexual abuse PN Physical neglect EN Emotional neglect

A series of multivariate regression models by incorporating five dimensions of resilience simultaneously revealed positive associations between SH and: (1) emotion regulation (OR = 0.39, 95% CI [0.26–0.58]) in PA; (2) goal concentration (OR = 0.39, 95% CI [0.29–0.53]), emotion regulation (OR = 0.35, 95% CI [0.20–0.60]) in EA; (3) goal concentration (OR = 0.52, 95% CI [0.35–0.75]), emotion regulation (OR: 0.37, 95% CI [0.19–0.71]) in SA; (4) goal concentration (OR = 0.67, 95% CI [0.48–0.94]), interpersonal assistance (OR = 0.49, 95% CI [0.33–0.72]), emotion regulation (OR = 0.42, 95% CI [0.27–0.64]) in PN; (5) interpersonal assistance (OR = 0.62; 95% CI [0.43–0.90]), emotion regulation (OR = 0.36, 95% CI [0.26–0.51]), and family support (OR = 0.63, 95% CI [0.49–0.82]) in EN.

### Resilience with SH repetition and severity in abused adolescents

**Figure 2:**
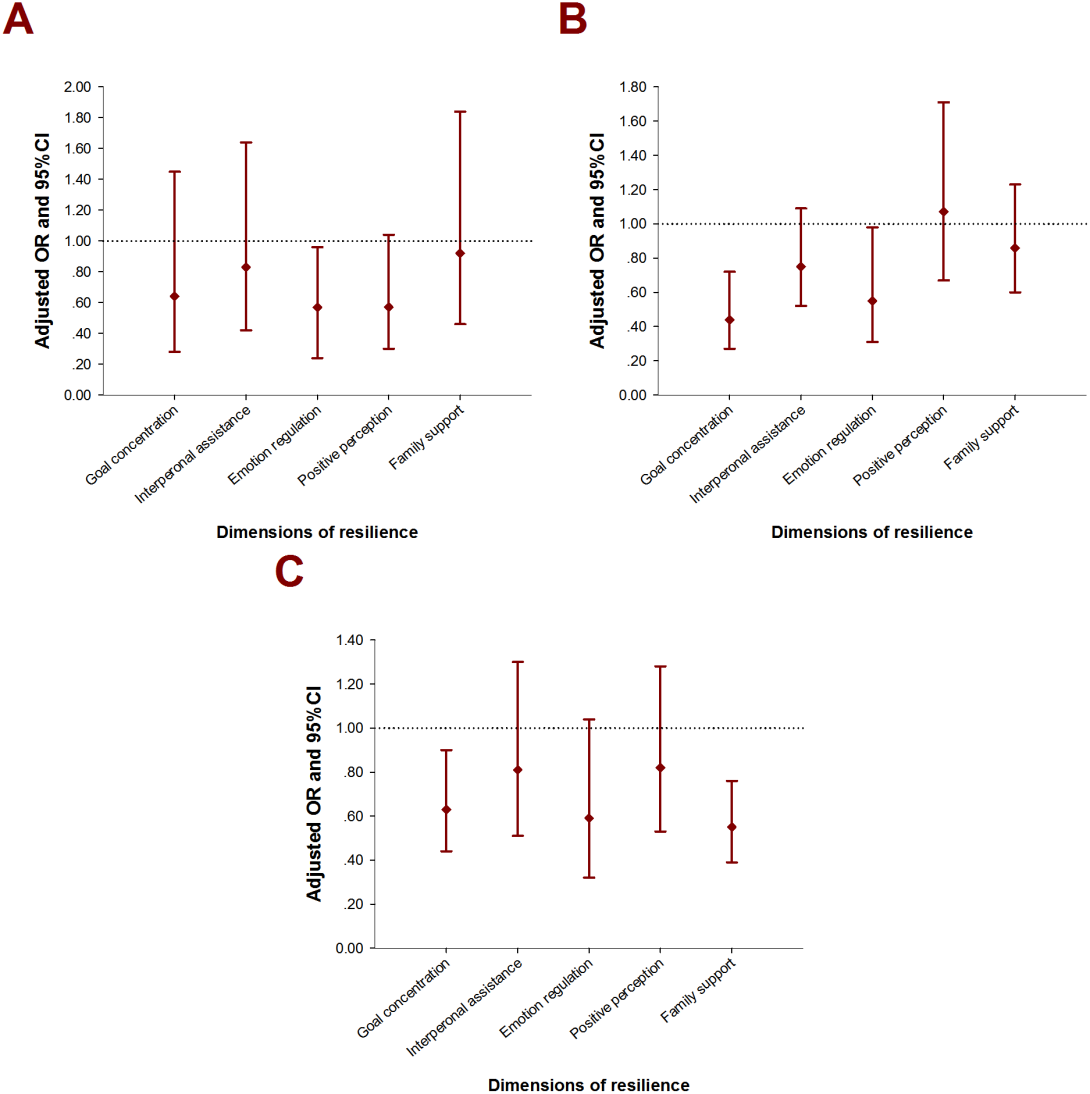
Adjusted OR with 95% CI for repeated SH by types of childhood maltreatment. (A) EA (Emotional abuse): adjusted for grade, whether boarding student, age of mother, alcohol drinking, bullying; (B) PN (Physical neglect): adjusted for age, grade, whether boarding student, whether single child, living situation, age of father, age of mother, alcohol drinking; (C) EN (Emotional neglect): adjusted for age, grade, whether boarding student, whether single child, living situation, age of mother, alcohol drinking, bullying.

**Figure 3:**
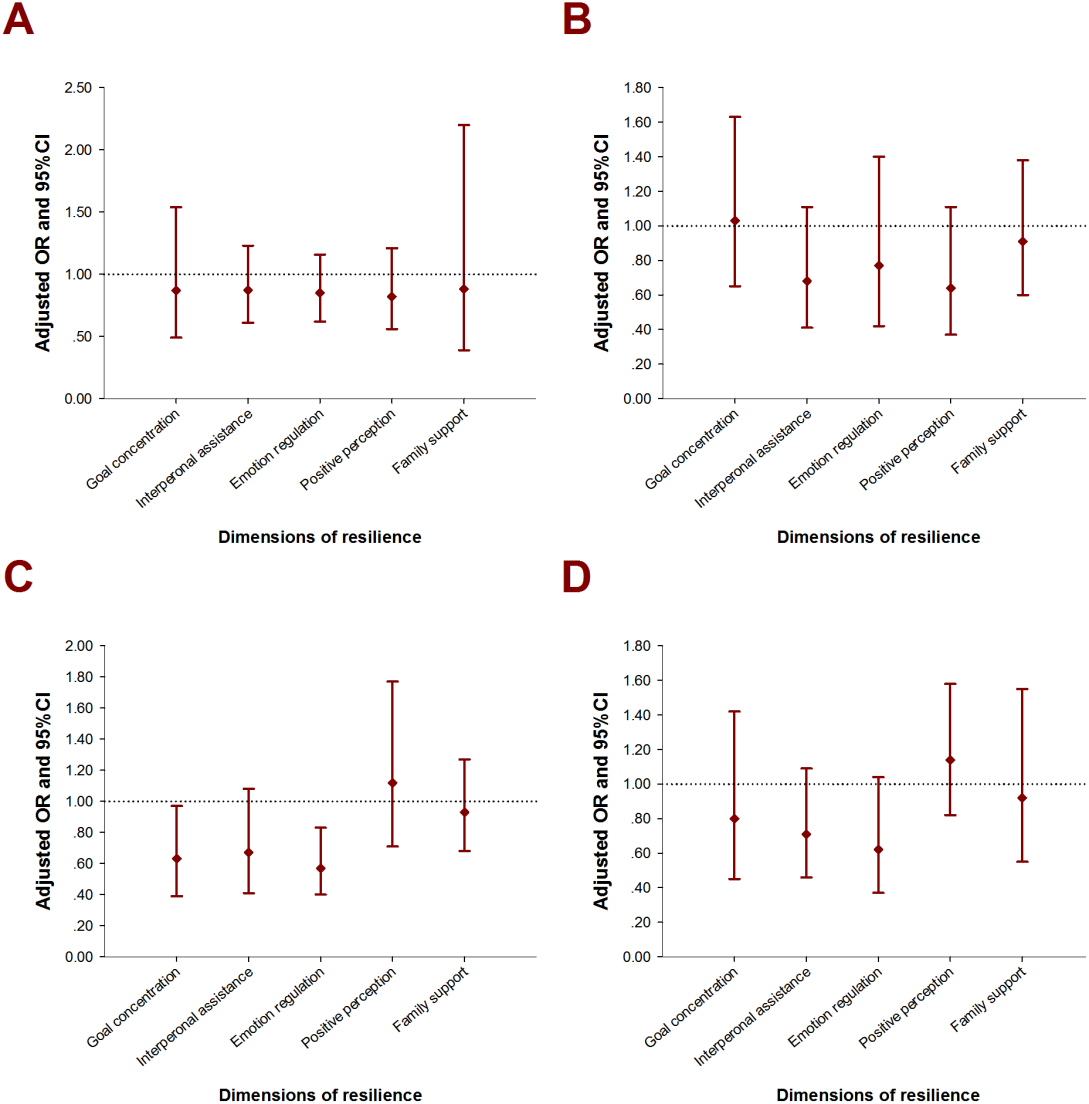
Adjusted OR with 95% CI for moderate to critical SH by types of childhood maltreatment. (A) EA (Emotional abuse): adjusted for grade, educational level of mother, alcohol drinking, bullying; (B) SA (Sexual abuse): adjusted for grade, alcohol drinking, bullying; (C) PN (Physical neglect): adjusted for grade, alcohol drinking, bullying; (D) EN (Emotional abuse): adjusted for grade, whether single child, alcohol drinking, bullying.

A subgroup Logistic regression among self-harmed subjects was performed with the intention to explore the associations between resilience and repeated and severe SH behaviors. For emotionally abused, as well as physically and emotionally neglected juveniles, the odds of repeated SH among more resilient children ranged from 0.39 (95% CI [0.23–0.66]) to 0.47 (95% CI [0.26–0.86]). With regard to the five dimensions of resilience, multivariate model suggested adverse associations between repetitive SH and goal concentration, emotion regulation, and family support in EA, PN, and EN (Fig. 2). By using the same strategy, the relationship between resilience dimensions and SH severity only presented significance in physically neglected groups: those who reported higher level in goal concentration, emotion regulation were related to ORs of 0.63 (95% CI [0.39–0.97]), 0.57 (95% CI [0.40–0.83]) in committing more severe SH (Fig. 3).

## Discussion

The present study investigated the hypothesis that psychological resilience serves as a protective factor against SH behavior in adolescents who had experienced childhood abuse. As expected, a predominant part of adolescents with childhood maltreatment history reported SH behaviors: 63.80%, 73.90%, 71.30%, 55.60%, and 58.20% for PA, EA, SA, PN, and EN. These prevalence rates are all significantly higher than an estimated SH prevalence of 30.30% in general Chinese adolescents (Xin et al., 2017). Besides, analytical results were supportive to our hypothesis that, resilience was inversely and independently associated with the prevalence, severity, and repetition of SH, and this inverse association varied among different types of childhood abuse.

Much in line with our anticipation, overall, resilience was significantly and negatively associated with the occurrence of SH in all abused subjects. Our findings are consistent with prior investigations which found that physical or emotional abuse exerted a disadvantageously impact on SH among middle school students (Paul & Ortin, 2019), and resilience potentially protected Chinese adolescents from hurting themselves (Yang et al., 2013; Ma, 2016), as self-injurers tended to be less resilient (Tian et al., 2019). The lifetime chance of suffering from depression, affective disorder, and borderline personality disorder (BPD) overtly increased among maltreated children (Anda et al., 2007; Herman, Perry & Van der Kolk, 1989), and these mental disorders could trigger more frequent SH behaviors (Hawton, Saunders & O’Connor, 2012). One reason behind this inverse association may be the fact that, exposure to domestic violence heightens the risk of developing psychological health problems, especially depressive symptoms (Gilbert et al., 2009), and resilience can substantially ameliorate the detrimental effect on mental health of being victims of abuse (Huang & Mossige, 2018). Other possible explanations involve dissociation, alexithymia, and self-blame, as suggested by previously published studies that predisposition to these disorders always come along with impaired resilience, thus rendering adolescents more vulnerable to negative life events (Swannell et al., 2012; Chaplo et al., 2015).

In view of the fact that parental neglect deprives children’s basic needs for love and care, neglected children can be well incorporated into the self-injury integrated model raised by Nock & Prinstein (2004). Under this situation, neglected adolescents may adopt SH behavior as a strategy to cope with distressed feeling of unwanted, unloved, and to shun intolerable family environment (Paul & Ortin, 2019). Taken together, by offsetting the detrimental effect of subsequent psychological problems that are at least partially induced by maltreatment experiences, resilience has the potential to convey protective effect on SH.

Further analysis found that, when it comes to the specific dimensions of resilience, emotion regulation appears to be the strongest factor against SH for all 5 types of childhood maltreatment. It has been reported that maltreated children and adolescents were more likely to present emotion regulation impairment, which finally gave rise to SH (Titelius et al., 2018). In the work of Shenk, Noll & Cassarly (2010), however, the mediation of psychological regulation did not reach a significant level in the linkage between domestic mistreatment and SH, which may be attributable to the different method used in determining child abuse by the authors. By impeding victims’ development of emotion regulation capabilities, child maltreatment elevates the risk of clinical psychopathological symptoms like depression, anxiety, and posttraumatic stress disorder (PTSD) (Shenk, Noll & Cassarly, 2010), which are established risk factors of SH (Jennissen et al., 2016). Therefore, individuals who had experienced childhood maltreatment may have difficulties in coping with emotional distress, which may lead to subsequent SH behaviors. In addition, Linehan’s theoretical model stresses that, growing in an environment that devalues emotion can incapacitate individual’s ability in correctly regulating feelings for adaptation (Linehan, 1993). As a result, to reconstruct emotion to a tolerable level, individuals may prefer strategies that are seen as temporary and impulsive, such as SH.

Another important finding is that, for adolescents who had experienced EA, SA, and PN, goal concentration, another dimension of resilience, was inversely associated with SH. Moreover, goal concentration was also inversely associated with repeated SH in PN and EN and severe SH in PN. Although still controversial, it has been suggested that attention problems are involved in the underlying mechanism between early domestic physical violence and SH (Paul & Ortin, 2019). Cumulative reports in the literature have linked attention deficit hyperactivity disorder (ADHD) with the development of SH via comorbid psychiatric problems. Of these ADHD symptoms, impulsivity and depression emerged as the most under-discussed mediators (Swanson, Owens & Hinshaw, 2014). Given that individuals with either concentration defect or ADHD have attention problems in common, and adolescents with abusive or neglectful parents are at higher risk of developing depression and impulsion (Eisenberg et al., 2004; Swanson, Owens & Hinshaw, 2014), we suspect that, the abused or neglected adolescents who are already psychologically vulnerable can exhibit reduced capacity in goal concentration, which largely presented in the form of depression or impulsivity, could upgrade their risk of SH as a way to avoid or counteract these distressful emotions. However, further studies are required to verify this assumption.

After effectively controlled for identified covariates, self-injuring adolescents who had experienced PN and EN reported fewer perceived interpersonal assistance. Existing evidence is supportive on the hazardous role of childhood abuse in the formation of secure attachment between victims and others (Roche, Runtz & Hunter, 1999; Davis, Petretic-Jackson & Ting, 2001). It has been known that social support can ameliorate the mentally destructive effect of negative events (Elklit, Pedersen & Jind, 2001). Therefore, it would be reasonable to suspect that, as an indispensable element of social support, interpersonal assistance can analogously buffer against the occurrence of SH caused by childhood maltreatment. Although a previous study by Wan et al. (2019) failed to conclude the positive association between interpersonal support and SI seems contradictory to our results, this discrepancy may lie in the different instruments used to measure interpersonal assistance. Future studies with the attempt to elaborately investigate this topic should be done.

The following limitations of our study should be noticed. To start with, the cross-sectional design inevitably prevents causal inference. Besides, cautions must be paid when extrapolating our results to other abused children or adolescents, as our study sample was drawn from a single county within Yunnan, China. Also, the retrospective measurement of childhood maltreatment by using CTQ can cause recall bias, which may influence the validity of study results. Despite these potential limitations, our study is the first exhaustive attempt to explore the associations between resilience and SH prevalence, repetition, and severity in maltreated Chinese adolescents by using large representative community sample. Our major findings are to be validated by future longitudinal studies in Chinese adolescents, or adolescents of other origins.

## Conclusions

In conclusion, this community-based cross-sectional study examined the protective role of psychological resilience, as well as its dimensions, on SH prevalence, repetition, and severity among abused Chinese adolescents. Results preliminarily evidenced that resilience in general was inversely associated with SH among abused adolescents. For specific dimensions of resilience, emotion regulation exhibited the strongest protective association with SH in all maltreated adolescents. Besides, interpersonal assistance and goal concentration were also found inversely associated with SH in adolescents who had experienced EA, SA, and PN. Our findings suggest the promising prospect of resilience-oriented intervention strategies and measures in preventing childhood maltreatment associated SH among adolescents, especially the measures which emphasize on improving emotion regulation ability, building goal concentration competency, and consolidating interpersonal assistance.

##  Supplemental Information

10.7717/peerj.9800/supp-1 Data S1 Data used for this study

10.7717/peerj.9800/supp-2 Table S1 Adjusted OR (95% CI) for self-harm by multivariable Logistic regression analysis among maltreated study subjects

## Additional Information and Declarations

### Data Availability

The following information was supplied regarding data availability: The raw data are available as Supplemental Files.
